# Cardioprotective Effects of Atorvastatin plus Trimetazidine in Percutaneous Coronary Intervention

**DOI:** 10.12669/pjms.292.2937

**Published:** 2013-04

**Authors:** Xuefeng Lin, Aiqun Ma, Wei Zhang, Qun Lu, Chaofeng Sun, Hongyan Tian, Xinjun Lei, Xiaojun Bai

**Affiliations:** 1 Xuefeng Lin, Department of Cardiovascular Medicine, First Affiliated Hospital of Medical College of Xi’an Jiaotong University; Institute of Cardiovascular Channelopathy, Key Laboratory of Environment & Genes Related to Diseases (Xi'an Jiaotong University), Ministry of Education; Key Laboratory of Molecular Cardiology, Shannxi Province; No.277 Yanta West Road, Xi’an, 710061, P. R. China.; 2 Aiqun Ma, Department of Cardiovascular Medicine, First Affiliated Hospital of Medical College of Xi’an Jiaotong University; Institute of Cardiovascular Channelopathy, Key Laboratory of Environment & Genes Related to Diseases (Xi'an Jiaotong University), Ministry of Education; Key Laboratory of Molecular Cardiology, Shannxi Province; No.277 Yanta West Road, Xi’an, 710061, P. R. China.; 3 Wei Zhang, Department of Cardiovascular Medicine, First Affiliated Hospital of Baotou Medical College, No.41 Linyin Road, Baotou, 014010, P. R. China.; 4 Qun Lu, Department of Cardiovascular Medicine, First Affiliated Hospital of Baotou Medical College, No.41 Linyin Road, Baotou, 014010, P. R. China.; 5 Chaofeng Sun, Department of Cardiovascular Medicine, First Affiliated Hospital of Baotou Medical College, No.41 Linyin Road, Baotou, 014010, P. R. China.; 6 Hongyan Tian, Department of Cardiovascular Medicine, First Affiliated Hospital of Baotou Medical College, No.41 Linyin Road, Baotou, 014010, P. R. China.; 7 Xinjun Lei, Department of Cardiovascular Medicine, First Affiliated Hospital of Baotou Medical College, No.41 Linyin Road, Baotou, 014010, P. R. China.; 8 Xiaojun Bai, Department of Cardiovascular Medicine, First Affiliated Hospital of Baotou Medical College, No.41 Linyin Road, Baotou, 014010, P. R. China.

**Keywords:** Trimetazidine, Atorvastatin, Percutaneous coronary intervention, Cardioprotection

## Abstract

***Objective:*** To explore the effects of preoperative administration of conventional doses of atorvastatin plus trimetazidine on the myocardial injury of patients during the perioperative period of percutaneous coronary intervention (PCI).

***Methodology: ***475 cases of acute coronary syndrome patients before PCI were randomly divided into the control group (238 cases) and experimental group (237 cases).The control group was treated with conventional doses of atorvastatin calcium (20 mg each time, once a night), and the experimental group was treated with conventional doses of atorvastatin calcium plus trimetazidine hydrochloride (20 mg each time, tid) for 3 d. After PCI, preoperative and postoperative 24 h concentrations of serum creatine kinase MB isoenzyme (CK-MB), cardiac troponin I (cTnI) and high sensitivity C-reactive protein (hs-CRP) as well as activity of myeloperoxidase (MPO) were investigated. Left ventricular ejection fractions of the patients were then examined 4 weeks later.

***Results: ***Postoperative 24 h cTnI concentration and elevated MPO activity of the experimental group were significantly lower than those of the control group (P <0 05). CK-MB activities and hs-CRP concentrations of the two groups did not differ significantly (P> 0 05).

***Conclusion: ***The administration of conventional doses of atorvastatin plus trimetazidine three days before PCI is able to protect the perioperative patients from myocardial injury.

## INTRODUCTION

Cardiovascular diseases, such as coronary heart disease, have become a prominent public health problem seriously threatening life expectancy and quality of life of people all over the world.^1^ Percutaneous coronary intervention (PCI) has been developing rapidly that primarily contributes to the revascularization of coronary heart disease patients. However, balloon dilatation and stent implantation operations during PCI will both induce myocardial ischemia and myocardial microembolization, which will lead to perioperative myocardial injury and affect therapy efficacy.^2^^,^^3^

ARMYDA-ACS study revealed that preoperative statins could reduce perioperative myocardial infarction (MI), but a large dosage of atorvastatin was used (80 mg/d),^4^ which may not be safely applied in the clinic treatment in China. It has been previously reported that atorvastatin plus trimetazidine could improve the symptoms of coronary heart disease and heart failure patients.

Thereby motivated, this study aims to investigate the effects of preoperative administration of conventional doses of atorvastatin plus trimetazidine on the myocardial injury of patients during the perioperative period of PCI.^5^

## METHODOLOGY

***Object:*** Seven hundred ninety six (796) patients were diagnosed as acute coronary syndrome and scheduled for coronary angiography from January 2010 to December 2011 in Department of Cardiovascular Internal Medicine of The First Affiliated Hospital of Medical College of Xi'an Jiaotong University. Four hundred seventy five patients that were going to have PCI surgeries were included in this study. Inclusion criteria: non- MI patients with normal baseline myocardial enzyme graphs, typical angina symptoms, positive treadmill test results as well as middle or severe lesion performance in at least one of coronary CT three-dimensional reconstruction. Besides, the following two requirements should be met simultaneously.^6^ 1) Single or multi-vessel lesions that have been confirmed by coronary angiography (vascular stenosis 70%) and were suitable for PCI; 2) patients who did not take trimetazidine in the last three months before hospitalization. Exclusion criteria: 1) obviously combined with liver and kidney dysfunctions, tumors, or other diseases such as myocarditis, heart failure and left ventricular ejection fraction <30% that may elevate creatine kinase-MB isoenzyme (CK-MB) activities and cardiac troponin I (cTnI) levels; 2) without combined diseases but with elevated preoperative baseline cTnI levels and CKMB activities; 3) Application of rotational ablation or directional coronary atherectomy; 4) intraoperative serious blood vessel complications and main branch obliteration, below grade 2 Thrombolysis in Myocardial Infarction (TIMI) confirmed by postoperative angiography; 5) patients who were allergic to trimetazidine.^7^ All the included patients have signed the informed consent forms. 475 patients were divided into trial group (odd, 238 cases) and control group (even, 237 cases) using a random number table.

***Apparatus*** **:** TDL-5-A centrifuge (Shanghai Anting Scientific Instrument Factory), AXIOM Artis dBc cardiac imaging equipment (Siemens AG, Germany), ACS180 automatic chemiluminescence immunoassay analyzer (for the detection of cTnI levels, Bayer, Germany), PRONTO Evolution automatic biochemical analyzer (Pentium, Italy) and CK-MB kit (Roche Diagnostics, Germany) were utilized. ELISA kits (Shanghai Gaochuang Chemical Technology Co., Ltd.) were utilized for the detection of human myeloperoxidase (MPO).

***Methods*** **:** Medical record data of all the selected patients were sampled, including smoking history, hypertension history, hyperlipidemia history, diabetes history and preoperative medication history, etc. Their body mass index (BMI) and left ventricular ejection fraction were also recorded.

MPO, high sensitivity C-reactive protein (hs-CRP), and cTnI levels before and after PCI were determined, perioperative MI incidences were counted (perioperative MI is defined as 99% of the upper limit of postoperative cTnI value more than 3 times of the reference value.^8^ On the preoperative 3rd day, patients in the control group were orally administered atorvastatin calcium (trade name Lipitor, Dalian Pfizer Pharmaceutical Co., Ltd.) (20 mg each time, once a night). Patients in the trial group were treated with trimetazidine hydrochloride (Nanjing Xin’gang Pharmaceutical Co., Ltd.) (20 mg each time, tid) besides equivalent doses of atorvastatin calcium. PCI procedures were performed according to routine operations. Angina symptoms induced by balloon dilatation and stent implantation during PCI as well as ST segment and T wave changes of their preoperative and postoperative electrocardiograms were recorded. Patients in the two groups were followed up within the postoperative 30 days, mainly including telephone follow-up of angina, MI, death, heart failure, re-hospitalization and re-intervention treatment and etc. Four weeks after PCI, ultrasound cardiograms of the patients were reexamined and left ventricular ejection fractions were recorded in the Outpatient Department.^9^

***Statistical analysis*** **:** All the statistics were independently processed and analyzed by a third party using SAS 9.1. All the data were expressed as mean ± s, comparison between the two groups was performed utilizing t-test or Wileoxon non-parametric test. Enumeration data were analyzed by Pearson chi-square test or Fisher's exact test. Two-sided tests were performed for all the statistics using P<0.05 as statistically significant.

## RESULTS

***Basic status of the two groups*** **: **In 238 cases of the control group, 156 cases were male, 121 cases and 40 cases suffered from hypertension and type 2 diabetes respectively, 39 cases were smokers, 52 cases orally administered angiotensin-converting enzyme inhibitors (ACEI), 32 cases were administered angiotensin receptor antagonists (ARB), and 28 cases were administered calcium channel blockers (CCB). Differences in the risk factors such as case number, gender, age and coronary heart disease, preoperative CK-MB activity, left ventricular ejection fraction, total cholesterol (TC), past medical history, and applications of ACEI, ARB, CCB of the two groups were not statistically significant. All the selected patients showed normal baseline cTnI levels, the mean values were less than 10 mg/L and did not affect the experimental results (Table-I).

***Comparison between PCI treatment effects of the two groups*** **:** Coronary artery lesion number, total time of balloon dilatation and stent implantation number of the two groups did not differ significantly (Table-II).

***Variations of myocardial injury markers and inflammatory markers*** **:** No significant differences were observed in the preoperative serum cTnI levels and CK-MB activities of the two groups. 24 h after PCI, cTnI levels of the trial group were significantly lower than those of the control group (P <0.05), whereas CK-MB activities of the two groups did not differ significantly (P>0.05). As shown in Table-III, increases in the postoperative MPO activities of the trial group were significantly lower than those of the control group (P <0.05), whereas postoperative hs-CRP levels of the two groups did not exhibit significant differences (P> 0.05).

**Table-I:** Basic status of the two groups

*Group*	*n*	*Age*	*CK-MB (zB/U/L )*	*Left ventricular ejection fraction (%)*	*TC (cB/mmol/L)*	*hs-CRP (ρB/mg/L)*	*MPO (cB/pmol/L)*
Control	238	68.6±7.2	12.5±0.4	60.1±4.1	4.8±0.2	3.4±0.7	226.9±12.8
Experiment	237	6.3±6.5	12.3±0.6	61.8±3.7	4.9±1.4	3.6±1.5	231.4±10.3

**Table-II:** PCI treatment effects of the two groups

*Group*	*n*	*Coronary artery lesion number*	*Total time of balloon dilatation (t/s)*	*Stent implantation number*
Control	238	1.75±0.63	43.1±4.4	1.8±0.6
Experiment	237	1.80±0.52	41.9±2.7	1.8±0.2

**Table-III:** Myocardial injury markers and inflammatory markers 24 h after PCI

*Group*	*n*	*cTnI (ρB/ng/ml)*	*CK-MB*	*△* * MPO*	*△* *hs-CRP*
Control	238	0.631±0.472	25.1±3.6	38.8±19.5	5.60±6.33
Experiment	237	0.384±0.527*	25.7±2.6	15.7±25.2*	5.29±8.17

* P< 0.05, compared to the control group.

## DISCUSSION

PCI techniques and equipment have been developing rapidly in interventional cardiology, and the success rates have already reached above 90% in most cardiac centers.^10^ However, PCI still frequently encounters perioperative myocardial injury, which together with inflammation have also been verified to be associated with long-term prognosis of patients. Currently, PCI-related regional myocardial injuries are mainly resulted from side branch occlusion, microsphere loss and transient myocardial ischemia that lead to the impaired blood perfusion of myocardial microcirculation.^11^ Bahrmann et al. have demonstrated that intracoronary microembolization during PCI was closely related to perioperative non-ST-segment elevation MI. Therefore, successful PCI is able to improve both the blood flow in epicardial great vessels and myocardial microcirculation.^12^

 Many studies have initiated the prevention or treatment of PCI perioperative myocardial injuries utilizing preoperative atorvastatin, trimetazidine, XZK, heart-protecting musk pills and etc. commonly. In particular, ARMYDA study published in 2004 showed that preoperative statins could reduce perioperative MI.^13^ Subsequently published ARMYDA-ACS study revealed that postoperative 30 d cardiac event incidence of the acute coronary syndrome patients who received high-dose atorvastatin (80 mg/d) in preoperative 12 h instead of other statins significantly reduced to 5%, whereas that of the control group was 17%. Naples II assay also obtained similar clinical outcomes utilizing the same dose of atorvastatin.^14^ Besides, post-hoc analysis showed that cardioprotective effects of statins were more obvious in the patients with higher baseline CRP levels. All the above phenomena are all ascribed to the pleiotropic effects of statins. In addition to lipid regulation, statins are also able to alleviate inflammation, improve endothelial function, reduce the expression of adhesion molecules, stabilize plaques, inhibit thrombus and etc. that all can protect myocardial cells.^15^

 Nevertheless, 80 mg statins were all adopted in the above clinical trials, which are too high for Chinese people and will thus hinder their clinical application due to security concerns. Main adverse reactions of statins include elevated liver enzymes and myopathy or even rhabdomyolysis. Although the reactions scarcely occur, they tend to depend on the dosage. Therefore, simultaneously reducing PCI-related myocardial injury and drug side effects have been widely spotlighted.^16^ In the present study, conventional doses of atorvastatin plus trimetazidine were employed, and the preliminary experimental results show that PCI postoperative myocardial injury marker cTnI and inflammatory marker of the trial group were all significantly lower than those of the control group, confirming that the combination therapy could effectively reduce myocardial injury.^17^^,^^18^ Owing to the relatively short follow-up time, left ventricular ejection fractions of the two groups did not differ significantly, which may be associated with the intrinsic mechanism of trimetazidine. As a common drug for the treatment of angina, trimetazidine transforms myocardial metabolism from the metabolism of fatty acids to the oxidation of glucose by primarily inhibiting the long-chain 3-Ketoacyl-CoA-thiolase in mitochondrion.^19^ As a result, trimetazidine can effectively control the energy supply equilibrium of free fatty acids or glucose oxidation, which thus can improve myocardial energy metabolism, reduce intracellular acidosis and calcium deposition and prevent cell membranes from oxygen free radical damages utilizing more generated ATP in the presence of limited oxygen during myocardial ischemia.^20^ Considering that follow-up time in this study was relatively short, perioperative MI still cannot be clearly demonstrated to be transformed into a certain medium-term or long-term benefit. In summary, this study provides a novel concept for reducing PCI perioperative MI, but further large-scale clinical study is still in need to determine a safe and effective perioperative myocardial protection program that is suitable for Chinese people.^21^
